# A rotator cuff sparing deltopectoral approach to intramedullary nail of humeral fractures

**DOI:** 10.1016/j.jseint.2026.101733

**Published:** 2026-05-19

**Authors:** Tom R. Doyle, Jessica M. Welch, Eoghan T. Hurley, Jay M. Levin, John R. Wickman, Christopher S. Klifto

**Affiliations:** a Department of Orthopaedic Surgery, Duke University Medical Center, Durham, North Carolina, USA; b Royal College of Surgeons in Ireland, Dublin, Ireland

**Keywords:** Humeral fracture, Anterograde nailing, Intramedullary nail, Deltopectoral, Rotator cuff sparing, Proximal humerus

## Abstract

**Background:**

Intramedullary nail (IMNs) for humeral fractures have been associated with iatrogenic rotator cuff damage resulting in poor shoulder function and pain. The purpose of this study was to evaluate nail positioning, clinical outcomes, and complications following IMN using a rotator cuff-sparing approach via the rotator interval.

**Methods:**

A retrospective review of patients who underwent an IMN for a humeral fracture with a minimum of 12 months follow-up at a single institution was carried out. Patients who underwent a mini-open deltopectoral approach utilizing the rotator interval were eligible. Clinical outcomes assessed included the American Shoulder and Elbow Surgeons, Single Assessment Numeric Evaluation, and visual analog scale scores, impingement, patient satisfaction, and range of motion (ROM). Nail positioning was assessed on radiographic follow up as were complications.

**Results:**

There were 22 patients available for radiological follow-up and 20 for clinical follow-up, of which 68.2% were female, the mean age was 67.7 ± 11.7 years, body mass index was 29.7 ± 8.8, and the mean follow-up was 25.9 ± 9.5 months. There were 7 proximal humerus fractures (4 II-part and 3 III-part fractures) and 15 fracture of the proximal shaft. The mean American Shoulder and Elbow Surgeons score was 87.9 ± 12.7, and the mean Single Assessment Numeric Evaluation score was 88.3 ± 12.8. The mean ROM in forward flexion was 149° ± 24°, abduction was 137° ± 27°, and external rotation was 47° ± 16°. There were no significant differences between fracture types for any outcome score or ROM. All nails were buried or flush with the humeral head on lateral x-rays the entry point was graded as central in 77% and anterior in 23% of patients. On AP x-rays, the entry point was graded as central in 87% of cases and lateral in 13%. The mean visual analog scale score was 0.7 ± 1.0, No patients reported persistent shoulder pain, and there were no revision or reoperations required. There was 1 case of delayed union with a broken locking screw and 1 case of adhesive capsulitis.

**Conclusion:**

Intramedullary nail of humeral fractures using a third generation nail and a rotator cuff sparing deltopectoral approach results in accurate nail positioning, a low risk of iatrogenic shoulder pain, as well as good ROM and functional outcomes; however, further comparative studies are required.

Humerus fractures are common, accounting for 7-8% of all adult fractures, with a bimodal distribution namely high energy trauma in younger patients and low energy mechanisms in older patients.^9^^,^^24^ While many proximal humerus and shaft fractures can be satisfactorily managed nonoperatively, a variety of surgical techniques have been developed.^17^^,^^36^ Open reduction with internal fixation using locking plates has been successfully employed; however, it is associated with large incisions, risks of symptomatic hardware, intraarticular screw penetration, varus collapse, and loss of fixation.^39^^,^^43^

Intramedullary nail (IMN) has emerged as an alternative technique, offering the potential for minimally invasive surgery, shorter operative times, as well as early motion, and return to function.^26^^,^^32^^,^^44^ It has also been associated with lower complication rates and time to union compared to open reduction with internal fixation.^10^^,^^38^ While comparable long-term functional scores and complication rates between the 2 group have reported.^10^ There is a greater body of level 1 evidence available for IMN of humeral shaft fractures, with no differences reported for nonunion, reoperation, or radial nerve injury, although IMN was associated with significantly quicker operative time and time to union, as well as lower rates of infection.^19^ No technique or approach to the humeral head has demonstrated clear superiority, and the choice is likely dependent on the fracture type and treating surgeon.^34^

Issues with IMN include complications such as impingement, rotator cuff tears, and damage to the shoulder joint or cartilage, which can lead to post-operative stiffness and pain.^15^^,^^42^ Previous studies suggesting shoulder pain and decreased range of motion (ROM) after IMN often involved early-generation implants and techniques that violates the rotator cuff.^1^ Newer IMN designs, which are straight, smaller in diameter and incorporate locked screws, have shown faster bone healing, and better functional outcomes.^12^^,^^27^ Rotator cuff-sparing techniques, including via the Neviaser and Mackenzie portals, can minimize iatrogenic shoulder pain, a problematic complication of antegrade humeral nailing.^1^^,^^6^ However, percutaneous reduction remains a technical challenge, without visualization of the cuff or of the fracture reduction.^14^^,^^23^ The deltopectoral approach for IMN has been described in the biomechanical and clinical literature as reliable method of reducing proximal humerus fractures involving the tuberosities and achieving a cuff sparing entry.^33^^,^^35^ The purpose of this study is to evaluate the clinical and radiological outcomes following IMN of proximal diaphyseal and proximal humerus fractures utilizing a rotator cuff-sparing technique via a deltopectoral approach. The hypothesis was that good functional outcomes and low complication rates would be observed with accurate nail insertion.

## Methods

### Patient selection

Ethical approval was sought and obtained from the local institutional review board; a retrospective review of prospectively collected data was carried out (JMW & TRD) to identify all patients who underwent intramedullary nail (IMN) of a humeral fracture with the senior author (CSK) between April 2019 and 2023. The operative notes of all patients who underwent an IMN for humeral fractures during this time were analyzed. Relative indications include: >50% fracture displacement of the surgical neck, ≥3 cm shortening, angulation >20° in the coronal or sagittal plane, and functional need for early weight bearing.^3^^,^^4^^,^^7^ The inclusion criteria for this study were: (1) adults ≥18 years, (2) undergoing IMN of a humeral fracture using a rotator cuff sparing deltopectoral incision, (3) 2, 3 or 4 part proximal humerus fractures and proximal 1/3 shaft fractures, (4) using a third-generation nail, and (5) and minimum 12 months follow-up. The exclusion criteria for this study were; (1) pathological, open and actively infected fractures (2) history of shoulder surgery or trauma (3) bilateral humeral fractures, (4) polytrauma or major neurovascular injuries, and (5) fractures >6 week old.

### Surgical technique

**Figure 1:**
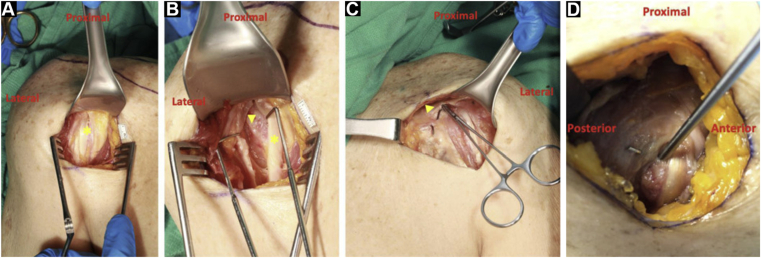
Cadaveric image of the deltopectoral approach for intramedullary nail fixation. (**A**) Deltopectoral incision, the biceps tendon within bicipital groove is marked with a ∗. (**B**) The biceps tendon is displaced medially, exposing the bicipital groove marked with ▼. (**C**) The coracohumeral ligament ◄ is partially released to expose the guidewire starting point. (**D**) Guide wire insertion.

All surgeries were performed under general anesthesia with interscalene block by a fellowship-trained shoulder surgeon (CSK). Patients were placed supine on a radiolucent table with the injured shoulder raised by 20-30°^.^ using a towel bump under the ipsilateral scapula. The proximal humerus was accessed through a deltopectoral approach with 4-cm-long incision extending from the coracoid process toward the midline of the humerus. A biceps tenodesis was performed to the pec major tendon with nondissolvable suture to limit the risk of symptomatic shoulder pain related to intra-articular biceps pathology. A partial release of the coracohumeral ligament with preservation of the coracoacromial ligament may be performed to improve access to the starting point as shown in Figure 1. The biceps tendon was transected proximal to the tenodesis and traced proximal to the rotator interval (RI) at the superior insertion of the subscapularis and anterior edge of the supraspinatus tendon. The biceps tendon was then transected at the origin of the superior labrum. A guidewire was placed through the center of the RI in an anteroposterior (AP) direction under fluoroscopic guidance. The shoulder was then extended approximately 15° to allow for access to the start point for the correct trajectory. The guidewire was directed medial to the footprint of the supraspinatus and 1.5 cm posterior to the bicipital groove nearing the apex of the humeral head. In fractures which involve the tuberosities, heavy nonabsorbable suture is placed in the rotator cuff to control the fragments, and they are provisionally reduced. When required, k-wire joysticks are used to augment reduction.

The wire was then directed in line with the humeral diaphysis, with entry point confirmed on AP, lateral, and axial fluoroscopic views, and is inserted into the proximal fragment as described by Saltzman et al.^35^ Radiographically, the ideal starting point through the RI is located at the medial aspect of the lateral third of the humeral head on the AP view and the posterior aspect of the anterior third of the humeral head on the lateral view.^35^ Both open and closed reduction of the fracture was performed using external manipulation and derotation of the humeral shaft, while the proximal fragment was manipulated through the incision with a starting guide, then the guide wire is passed across the fracture into the intramedullary canal of the distal fragment. A system-specific entry reamer was used to open the cortex of the humerus proximally. A radiographic ruler is used to estimate the appropriate size nail. If required to accommodate the diameter of the nail distally, the canal can then be serially reamed until the point of chatter, and the length is measured over the guidewire. Next, a third-generation intramedullary nail is passed over the guidewire from proximally to distally until the head of the nail was buried 1 cm within the proximal humerus (Polaris 3, Acumed, Beaverton, OR, USA, Trigen proximal straight humeral nail, Smith & Nephew, Watford, UK or Tornier Aequalis intramedullary nail, Wright Medical, Memphis, TN, USA). Interlocking screws are placed proximally and then distally. The number of proximal interlocking screws used was dictated by the fracture pattern and bone quality, typically ranging from 3 to 5 screws. Screws were placed through the open incision. Multiplanar fluoroscopy was used to assess fracture reduction and to assess proximal humerus screw length to confirm there was no penetration of the articular surface. The RI was closed with size 0 absorbable braided suture. The remaining layers were then closed in standard fashion.

### Rehabilitation protocol

Post-operatively, patients were immobilized in a sling until the regional anesthetic resolved, they are then allowed return to activities of daily living. Physical therapy is initiated 2 weeks post-operatively and patients progress through passive, assisted, active ROM exercises followed by strengthening exercises after 6 weeks.

### Follow-up and assessment

Patients routinely presented for post-operative follow-up at 2, 6, 12 weeks; 3 and 6 months; and at 1 year. Clinical outcomes were either assessed at the final visit. ROM, including forward flexion, abduction, external rotation, and internal rotation (IR) was recorded with a goniometer by a single physician associate blinded to the treatment. IR was measured using the Flurin et al method.^16^ In IR, the highest vertebral level reached by the thumb was scored as: 0° = 0, hip = 1, buttocks = 2, sacrum = 3, L5-L4 = 4, L3-L1 = 5, T12-T8 = 6, and ≥T7 = 7. Patient-reported outcomes measures were collected including American Shoulder and Elbow Surgeons (ASES) score, Single Assessment Numeric Evaluation (SANE), visual analog scale (VAS) pain, and Patient-Reported Outcome Measurement Information System (PROMIS) Upper Extremity and Physical Function scores. Complications including hardware issues, reoperations, persistent shoulder pain, and restricted ROM were also noted. Impingement was defined as symptoms of pain with overhead activity, in patients with consistent clinical examination and imaging findings.^8^

All radiographic assessments were performed by 2 independent researchers who were not involved in the patients' care and were blinded to the final outcome (TRD & ETH), the senior author was nominated to resolve any disagreement. Fractures were classified according to the Arbeitsgemeinschaft für Osteosynthesefragen/Orthopedic Trauma Association classification, and proximal humeral fractures were also classified using the Neer classification.^26^^,^^30^ At each post-operative visit, radiographs were taken. X-rays were reviewed for hardware complications to assess for fracture union and for avascular necrosis of the humeral head. Nonunion was defined as the absence of bridging callus for at least 3 cortices at 6 months follow-up. X-rays were reviewed to assess the nail entry point. AP images were used to grade entry as medial, centered, or lateral and lateral images were used to grade entry as anterior, centered, or posterior.^2^ Nails were considered flush if < 1 cm from humeral epiphyseal line, buried if > 1 cm, and proud if extending proximal to the articular margin.

### Statistical analysis

Statistical analysis was performed using Stata (Stata corp 2024). For all continuous and categorical variables, descriptive statistics were calculated. Continuous variables were reported as weighted mean and standard deviation, and were analyzed using either the Student *t* test or Mann–Whitney *U* test, whereas categorical variables were reported as frequencies with percentages and were analyzed using Fischer exact test. *P* values < .05 were considered statistically significant.

## Results

### Patient demographics

During the study period, there were 31 potentially eligible patients. There were 5 patients (16.1%) who died of unrelated causes between 5 and 8 months post-operatively, all of which showed radiographic evidence of healing at their final review. Four patients (12.9%) were lost to follow-up. There were 22 patients available with complete radiographic follow-up, 2 patients were excluded from the functional outcome scores on the basis of severe cognitive impairment, and ipsilateral hemiparesis predating the injury.

The mean age was 67.7 ± 11.7 years (range: 34-93), mean follow-up was 25.9 ± 9.5 months (12-41 months) and mean body mass index was 29.7 ± 8.8 as shown in Table I. There were 15 females (68.2%), 4 smokers (18.2%), and 9 patients (40.9%) with diabetes. There were 12 patients (54.5%) who had fracture fixation of their dominant limb. Just 1 patient (4.5%) had a high-energy mechanism of injury, with the remained occurring from standing height falls. There were 7 fractures of the proximal humerus and 15 fractures of proximal third of the humeral shaft. Using the Neer classification, there were 4 II-part fractures and 3 III-part fractures of the proximal humerus. The Arbeitsgemeinschaft für Osteosynthesefragen classification of the fractures is shown in Table II.

**Table I:** Patient demographics.

Variable	Mean ± SD
Age	67.7 ± 11.7
Months follow-up	25.9 ± 9.5
Female, n (%)	15 (68.2%)
BMI	29.7 ± 8.8
Injury to dominant arm, n (%)	12 (54.7%)

*SD*, standard deviation; *n*, number; *BMI*, body mass index.

**Table II:** Fracture classification.

AO fracture classification	N	%
11A2	9	29.0
11B1.1	4	12.9
12A1	10	32.3
12A3	1	3.2
12B2	5	16.1
12B3	2	6.5

*AO*, Arbeitsgemeinschaft für Osteosynthesefragen.

The mean operative time was 130 ± 33 minutes. There were 8 short nails (36.3%) and 14 long nails (63.6%). All proximal humerus fractures received short nails as did 1 fracture of the proximal diaphysis, while the remainder received long nails. There was no significant difference in operative time between long and short nails (134 ± 25 vs. ± 123 ± 45 minutes, *P* = .467).

### Functional outcomes

The mean range of forward flexion at final follow-up was 149° ± 24°, abduction of 137° ± 27°, external rotation of 47° ± 16°, and IR of 5 ± 1. The mean shoulder SANE score was 88.3 ± 12.8, ASES score was 87.9 ± 12.7, VAS pain score 0.7 ± 1.0, PROMIS upper extremity function 34.9 ± 6.9, and PROMIS physical function score was 41.5 ± 7.6, as shown in Table III. All patients were either satisfied or very satisfied. There were no significant differences for any functional outcome between those who had surgery on their dominant limb vs. nondominant limb or between proximal humerus vs. proximal shaft fractures (*P* > .05 for all).

**Table III:** Patient-reported outcomes measures and range of motion.

Variable	Mean ± SD
Range of Motion	
Flexion	149° ± 24°
Abduction	137° ± 27°
External rotation	47° ± 16°
Internal rotation	5 ± 1
PROM	
VAS	0.7 ± 1.0
SANE	88.3 ± 12.8
ASES	87.9 ± 12.7
PROMIS UEF	34.9 ± 6.9
PROMIS PF	41.5 ± 7.6

*SD*, standard deviation; *VAS*, visual analog scale; *SANE*, Single Assessment Numerical Evaluation; *ASES*, American Shoulder Elbow Surgeons; *PROM*, patient-reported outcome measure; *PROMIS*, Patient-Reported Outcomes Measurement Information System; *UEF*, upper extremity function; *PF*, physical function.

### Radiographic findings and complications

Radiographic assessment was performed on all 31 patients. On AP x-rays the nail entry point was graded as central in 27 patients (87.1%) and lateral in 4 patients (12.9%). On lateral x-rays, the nail entry point was graded as central in 24 patients (77.4%) and anterior in 7 patients (22.7%). There were 20 nails (64.5%) with a center/center position. There were 15 nails that were graded as flush (48.4%) and 16 which were buried (51.6%), and none that were proud, as shown in Table IV.

**Table IV:** Radiographic findings.

Variable	N (%)
AP view	
Lateral	4 (12.9%)
Central	27 (87.1%)
Medial	0
Lateral view	
Posterior	0
Central	24 (77.4%)
Anterior	7 (22.6%)
Nail position	
Proud	0
Flush	15 (48.4%)
Buried	16 (51.6%)

*AP*, anteroposterior; *N*, number.

**Figure 2:**
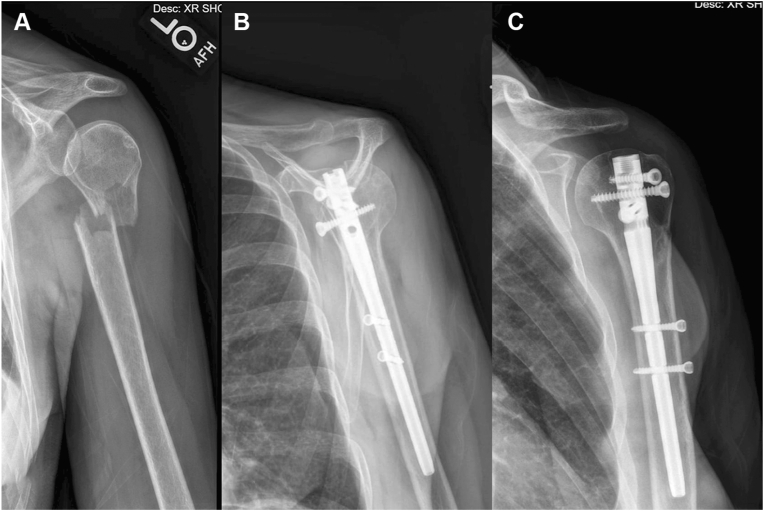
X-ray showing (**A**) a fracture of the surgical neck of the humerus, (**B**) scapular Y and (**C**) AP views showing intramedullary nail fixation. *AP*, anteroposterior

There were no fractures through the anatomic neck of the humerus or articular surface head-splitting fractures. No patients went to develop osteonecrosis of the humeral head. A loss of fracture reduction or tuberosity escape was not observed in any patients, example shown in Figure 2. There was one case of delayed union in a female, with risk factors included age >90 years, rheumatoid arthritis, former smoker, medial hinge disruption, and <8 mm of calcar length who went on to heal at 12 months. The most proximal locking screw in this case broke but was not causing harm. The patient was satisfied with her condition, and no intervention was attempted. There were no other hardware complications or revision operations. There was 1 case of adhesive capsulitis, with the patient undergoing capsular release at another institution, and 2 cases of post-operative shoulder pain, both patients received intra-articular steroid injections, and their pain improved in time (VAS of 1, for both). At final follow-up, there were no patients who experienced impingement symptoms.

## Discussion

The most important findings of this study is that intramedullary nail of humeral fractures using a third-generation nail and a rotator cuff spearing deltopectoral approach results in accurate nail entry and leads to good ROM, functional outcomes, and fracture healing. Persistent shoulder pain and poor ROM are commonly reported after traditional approaches to IMN of the humerus, although these complications appear to be minimized with the cuff sparing approach through the RI with routine biceps tenodesis.

Historically, the nails used for IMN of the humerus were “bent” to avoid the articular cartilage of the humeral head; however, they were associated with complications such as varus alignment, iatrogenic fractures of the greater tuberosity, and injury to the rotator cuff.^13^^,^^25^^,^^40^ The resultant lateral entry point commonly led to rotator cuff tendon violation causing chronic shoulder pain, poor ROM, and functional outcomes.^20^^,^^27^ This lead to the development of “straight” nail designs, which allowed a more medial entry point, offering increased biomechanical strength and a reduced likelihood of cuff tendon damage.^20^^,^^21^^,^^31^ However, despite these improvements concerns have persisted regarding rotator cuff tendon related complications and an over medialized entry point which crosses articular humeral cartilage.^22^ This led to a search for alternatives to the traditional deltoid splitting approach to improve the starting point and clinical outcomes.^12^ IMN through the portal of Neviaser, which can be performed percutaneously or open has emerged as an option for both proximal humerus and shaft fractures, with promising initial results^5^^,^^11^^,^^18^^,^^41^ While it has been argued performing IMN in the lateral position is easier,^2^ Boileau et al have proposed various percutaneous portals for IMN of the surgical neck of humerus fractures based on the displacement of the fracture, with a portal anterolateral to the acromion for valgus head fracture angulation, a portal anterior to the acromion for fractures with shaft translation but no proximal fracture angulation, a portal anterior to the acromioclavicular joint for varus head fracture angulation, and a Neviaser portal for posterior tilt and varus fracture angulation of the proximal fragment.^5^ This varied approach has several advantages, as it is percutaneous and offers an optimized trajectory to reduce the fracture. However, this level of variation may be a challenge for surgeons in creating a reproducible surgical method and thus lead to inconsistent humeral entry points.

There is a paucity of available clinical or biomechanical research regarding the deltopectoral approach to IMN.^33^^,^^35^ The benefits of the min-open deltopectoral incision described in this study include a single reproducible surgical approach for all fracture types, with reliable IMN center/center humeral head position. There were no cases of posterior or medial nail entry observed, while just 4 nails (12.9%) had a lateral entry point, which has previously been associated with greater cuff violation and inferior screw fixation of the tuberosities.^20^ In addition, there were no nails, which were proud above the humeral epiphysis. The mini-open incision resulted in a consistent cuff tendon sparing entry through the RI. Although we do not have radiological confirmation of the status of the cuff at final follow-up, the clinical implications are clear with all patients being either satisfied or very satisfied, no patients reporting persistent shoulder pain or impingement symptoms and a mean VAS score <1. This compares favorably to the literature with rotator cuff symptoms reported in 6% of patients after third-generation IMN.^22^ Similarly, there was excellent preservation of shoulder ROM, with the mean values exceeding those required for a functional ROM and close to the normative values for what is a predominantly elderly comorbid population.^14^^,^^29^ The patient-reported outcomes measure scores also showed good shoulder function, with a mean SANE score of 88.3 ± 12.8 and ASES score of 87.9 ± 12.7, with no differences observed between dominant and nondominant limbs. Boileau et al have previously argued that entering the cuff musculature with a 9-mm third-generation IMN is effectively equivalent to the standard practice of inserting an 8-mm cannula during shoulder arthroscopy.^5^ The clinical results presented in this study support this hypothesis and confirm that the deltopectoral mini-open approach to anterograde IMN of the humerus results in a safe and reproducible operation with low rates of cuff related complications.

IMN has several advantages for fracture healing compared to open plate fixation, with closed reduction allowing for preservation of the fracture hematoma and vascular supply to the fragments, while smaller incisions and quicker operating time likely contribute to reduced rates of infection.^19^^,^^27^ There was a low rate of complications observed using this technique, with a mean follow-up over two years. There were no post-operative infections observed in keeping with the reported rate of 1% after IMN and substantially lower than the reported rate of 5% after open fixation.^19^ However, poor function and pain after IMN fixation have previously been reported even in patients with radiologically confirmed intact rotator cuffs.^42^ Muccioli & Boileau have reported symptomatic long head of biceps (LHBs) pathology in 20% of patients after percutaneous IMN, resulting in significant functional impairments, while cuff tears did not have a statistically significant effect.^28^ The LHB tendon can be injured by the fracture, proximal locking screws insertion, or proud seating, while the LHB may become incarcerated in scar tissue in the bicipital groove or in the glenohumeral joint.^8^^,^^28^^,^^37^^,^^42^ In addition, degenerative LHB tendons may be observed in older patients. Christ et al have previously described a technique of routine biceps tenodesis during IMN performed via an anterolateral acromial approach, with positive pain and functional post-operative outcomes.^8^ In our study, routine biceps tenodesis was performed, and there were no resultant complications observed. While biceps tenodesis may not be mandatory, positive outcomes are reported in this series with low pain scores and rates of impingement. There was 1 case (4.5%) of asymptomatic delayed union observed in a patient with notable risk factors including advanced age and rheumatoid arthritis. There were no cases of nonunion, although rates of 5-8% have been reported elsewhere.^45^ The deltopectoral rotator cuff-sparing IMN approach appears to be a safe operation that minimizes complications.

### Limitations

Limitations of this study include the small size, the heterogeneity of the included fractures and nails, and a lack of magnetic resonance imaging assessment of the post-operative cuff integrity. There is no control group available for comparison, but we no longer offer plate fixation to patients suitable for IMN. Further research comparing this approach to other modern nailing techniques will be required. As this is a trauma series, no pre-operative functional scores are available for comparison. It also may be considered a limitation that this is a single surgeon series.

## Conclusion

Intramedullary nail of humeral fractures using a third-generation nail and a rotator cuff-sparing deltopectoral approach results in accurate nail positioning, a low risk of iatrogenic shoulder pain, as well as good ROM and functional outcomes; however, further comparative studies are required.

## Disclaimers:

Funding: No funding was disclosed by the authors.

Conflicts of interest: Jay M Levin reports a relationship with Stryker that includes: Stock or stock Options. He also reports a relationship with Zimmer that includes: Stock or stock Options.

Christopher S Klifto reports a relationship with Acumed LLC that includes: Paid consultant, He also reports a relationship with GE healthcare that includes: Stock or stock Options, Christopher S Klifto reports a relationship with Johnson & Johnson that includes: Stock or stock Options, He aslo reports a relationship with Merck that includes: Stock or stock Options, Christopher S Klifto reports a relationship Pfizer with that includes: Stock or stock Options, He also reports a relationship with Resore 3D that includes: Paid consultant, Christopher S Klifto reports a relationship with Smith & Nephew that includes: Paid consultant.

Any additional authors, their immediate families, and any research foundations with which they are affiliated have not received any financial payments or other benefits from any commercial entity related to the subject of this article.
